# Exploring internalized stigma among people living with psychosis and their caregivers: Insights from a community rehabilitation initiative in Malawi

**DOI:** 10.1371/journal.pmen.0000753

**Published:** 2026-11-05

**Authors:** Mia Buono, Kazione Kulisewa, Brian W. Pence, Bradley N. Gaynes, Abigail M. Morrison, Joshua Chienda, Melissa A. Stockton

**Affiliations:** 1 Department of Epidemiology, Gillings School of Global Public Health, University of North Carolina at Chapel Hill, Chapel Hill, North Carolina, United States of America; 2 Department of Psychiatry and Mental Health, Kamuzu University of Health Sciences, Blantyre, Malawi; 3 Department of Psychiatry, University of North Carolina at Chapel Hill School of Medicine, Chapel Hill, North Carolina, United States of America; 4 Department of Health Behavior, Gillings School of Global Public Health, University of North Carolina at Chapel Hill, Chapel Hill, North Carolina, United States of America; 5 Psychiatry Department, Perelman School of Medicine, University of Pennsylvania, Philadelphia, Pennsylvania, United States of America; American University of Beirut, LEBANON

## Abstract

People living with psychosis (PWLE) experience, anticipate, and internalize stigma. Over time, experienced and anticipated stigma can increase internalized stigma, negatively impacting health. However, predictors and outcomes of internalized stigma among PWLE and their caregivers remain unknown. We explore how stigma changes over time; the associations between hypothesized predictors and internalized stigma; and how internalized stigma influences mental health and functional outcomes. We analyzed data from a two-arm pilot randomized controlled trial comparing a community-based rehabilitation intervention to enhanced usual care in Blantyre, Malawi (NCT06080477). Sixty PWLE and 118 caregivers who met eligibility criteria completed interviewer-administered surveys at baseline, 6, and 12 months. Exploratory factor analysis (EFA) assessed the dimensionality of stigma scales. We used correlations and adjusted generalized estimating equations to examine associations between predictors and outcomes of internalized stigma. EFA supported single-factor structures with adequate internal consistency for most stigma scales. Among PWLE, mean internalized stigma, anticipated stigma, and experienced stigma decreased from baseline to 12 months in both arms. Stigma measures were generally comparable between arms except for family-enacted stigma, which was lower at 12 months among those receiving the intervention compared to usual care (mean difference = -0.97; -1.88, -0.05). At baseline, higher family-enacted stigma was moderately associated with higher internalized stigma scores (correlation coefficient = 0.34; 0.13, 0.53). Greater change in family-enacted stigma (regression coefficient = 0.24; 0.04, 0.43) and anticipated stigma (0.14; 0.02, 0.26) were weakly associated with greater change in internalized stigma. At baseline, internalized stigma was weakly associated with depressive (0.14; -0.08, 0.35) and anxiety symptoms (0.11; -0.08, 0.31). Over time, greater increases in internalized stigma were associated with higher anxiety (0.13; 0.01, 0.24). Caregivers’ internalized stigma decreased over 12 months, with no associated predictors. Anticipated, experienced, and internalized stigma are prevalent among PWLE in Malawi. Findings suggest the need to continue to explore the mechanisms between predictors and outcomes of internalized stigma.

## Introduction

Schizophrenia is the third largest contributor to global disability, highlighting its profound impact on quality of life and functionality [1]. Individuals living with psychosis experience a high burden of stigma [2,3]. Stigma in this population can manifest through experienced stigma (direct enacted mistreatment, discrimination, or societal exclusion), anticipated stigma (anticipated or feared mistreatment or discrimination), and internalized stigma (accepting negative beliefs about oneself) [4,5]. These forms of stigma are often perpetuated through societal stereotyping and labeling of psychotic symptoms as dangerous and unpredictable [6]. This labeling leads health care providers, community members, and family members to interact with people with lived experience (PWLE) of psychosis in differential or negative ways through stereotyping, discriminatory behavior, and prejudices [6]. In addition to perpetuating stigma, family members of PWLE may also be subject to discriminatory behavior and prejudice through association with PWLE [7–10]. While evidence is limited, family members experiencing secondary stigma may treat their relatives with more stigmatizing behaviors such as social distancing and concealment [9,11]. Over time, more experienced and anticipated stigma can lead to the internalization or adoption of negative beliefs about oneself, increasing internalized stigma among PWLE [2,12,13]. The internalization of such beliefs has been shown to reduce health-seeking behaviors [2,14–16] and negatively influence mental health and functional outcomes among people with psychosis [12,17–19].

While the effects and mechanisms of stigma among PWLE and their family members have been explored in high-income contexts, little work has been done in low- and middle-income countries (LMICs). In Malawi, a country with a high psychosis burden [20,21], research has shown that stigmatizing beliefs and behaviors about psychosis are widespread. Psychosis is often attributed to drug use, spiritual possession, witchcraft, and curses for sins in Malawi [22]. The main drivers of stigma in this region include limited knowledge, negative stereotypes and fear, and blame placed on those with the disorder [23–25]. Interventions that address these drivers through education, involvement of the community, and a focus on rehabilitation [26–29] hold promise in changing community attitudes and reducing experienced, anticipated, and subsequently internalized stigma [3,15,30]. However, little is known about predictors and outcomes of internalized stigma and the prevalence of various forms of stigma over time among PWLE and caregivers in low-income countries such as Malawi.

Therefore, we address this gap by examining internalized stigma among PWLE and caregivers participating in a two-arm pilot trial comparing a community-based rehabilitation (CBR) intervention to enhanced usual care in Malawi. We explore how experienced, anticipated, and internalized stigma change over the course of the study period; the associations between hypothesized predictors and internalized stigma; and how internalized stigma influences mental health and functional outcomes.

## Methods

### Ethics statement

The study was approved by the institutional review boards at the University of North Carolina at Chapel Hill and the Malawi National Health Sciences Research Committee (NHSRC), under the Ministry of Health (MOH). This trial was registered at ClinicalTrials.gov before enrollment (NCT06080477). The authors had access to information that could identify individual participants during and after data collection. Additional information regarding ethical, cultural, and scientific considerations specific to inclusivity in global research is included in the Supporting Information (S1 Checklist).

**Fig 1:**
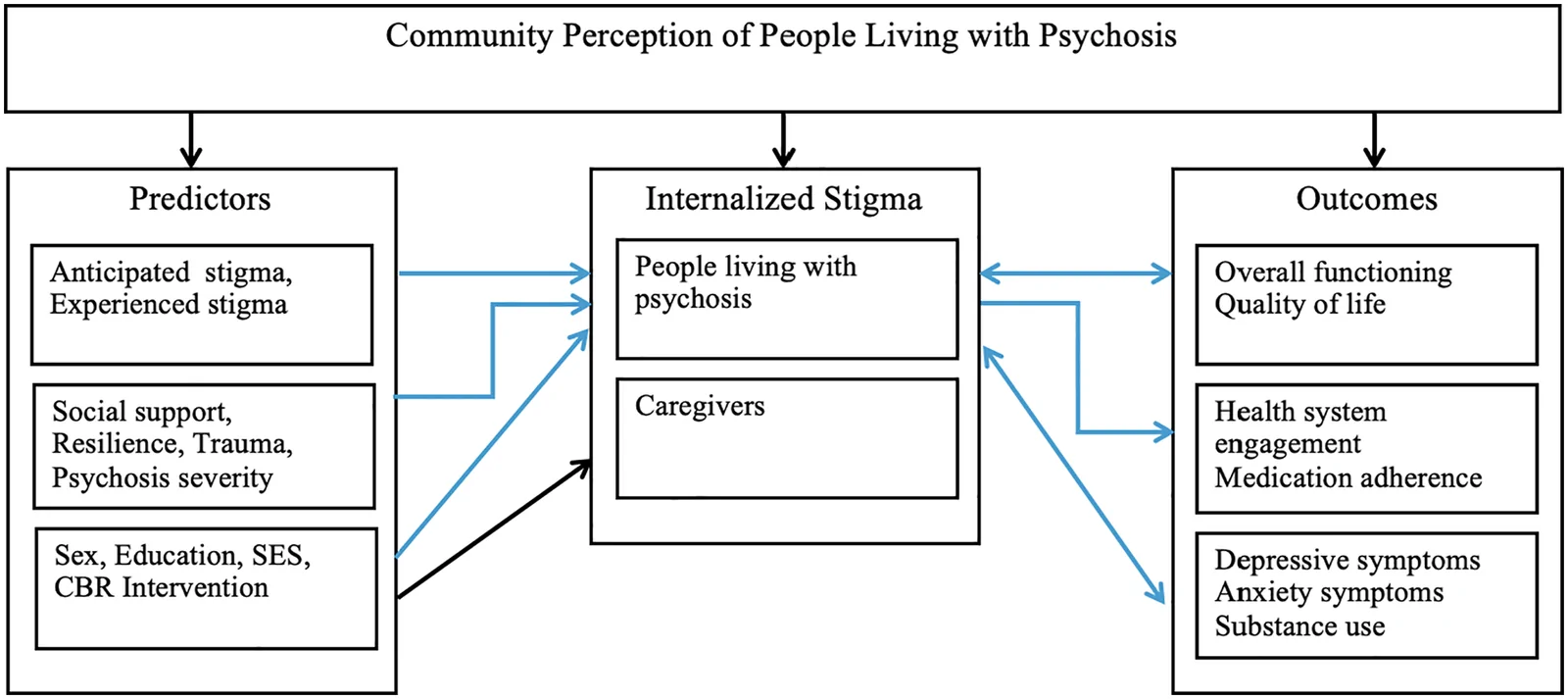
Conceptual framework of predictors and outcomes of internalized stigma among people living with psychosis and their caregivers in Malawi. Blue lines represent predictor and outcome pathways among PWLE. Black lines represent predictor pathways among caregivers. Measures: Anticipated stigma: Discrimination and Stigma Subscale (DISC-12 subscale); Experienced Stigma: DISC-12 subscale; Resilience: DISC-12 subscale; Psychosis Severity: Positive and Negative Syndrome Scale (PANSS); Sex, Education, Socioeconomic Status (SES): Demographic Survey; Community-Based Rehabilitation (CBR) Intervention: Study arm; Internalized stigma: 10-Item Internalized Stigma of Mental Illness Inventory (ISMI-10) (PWLE), 17-Item Parents’ Internalized Stigma of Mental Illness Inventory (PISMI) (caregivers); Overall functioning: 12-item World Health Organization Disability Assessment Schedule (WHODAS); Quality of life: Short Form-8 Health Survey (SF-8); Depressive symptoms: Patient Health Questionnaire- 9 (PHQ-9); Anxiety symptoms: Generalized Anxiety Disorder-7 Scale (GAD-7).

### Conceptual framework

The conceptual model guiding this paper (Fig 1) hypothesizes that among people with lived experience and their caregivers in Malawi, anticipated and experienced stigma, in addition to other demographic [31] and psychosocial factors [32,33], will predict levels of internalized stigma at baseline and follow-up. We hypothesize that internalized stigma in turn affects subsequent functioning [34], quality of life [35,36], and mental health [16,17]. This framework guided the selection of variables and the analytical approach for examining predictors of internalized stigma, changes in internalized stigma, and the relationship of changes in internalized stigma to changes in hypothesized outcomes over the study period.

### Design

This study is a post hoc secondary analysis of data from a two-arm pilot randomized controlled trial that compared an adapted CBR intervention to enhanced usual care among community-dwelling individuals with lived experience of psychosis and their caregivers in Malawi (NCT06080477). This analysis focuses on internalized stigma, an outcome that was not a primary outcome of the parent trial, and explores how stigma changed over the course of the study period; the associations between hypothesized predictors and internalized stigma; and how internalized stigma influences mental health and functional outcomes.

### Participants

Participants were recruited from the Queen Elizabeth Central Hospital (QECH) in Blantyre, Malawi, from December 5^th^, 2023, to July 30^th^, 2024. Participants attending outpatient services who met the following eligibility criteria were included in the study: 1) were 18 years or older, 2) had a diagnosis of schizophrenia spectrum disorder or clinical presentation of symptoms of hallucinations, delusions or thought disorder that persisted for longer than one month accompanied by significant functional impairment, 3) were a Blantyre District resident, 4) were not planning to relocate out of Blantyre District in the next 12 months, 5) had a primary caregiver willing to participate in the study, and 6) had current elevated symptoms of poor functioning as demonstrated by one or more of the following: Positive and Negative Symptoms Scale score ≥58, 12-item WHO Disability Assessment Schedule 2.0 score ≥35, and/or Clinical Global Impression Severity score ≥3 (at least mildly ill).

Up to two caregivers per participant were recruited to participate to assess the preliminary feasibility and acceptability of the trial. All enrolled caregivers met the following eligibility criteria: 1) a current caregiver for an eligible and consenting patient participant, 2) 18 years or older, 3) a Blantyre District resident, and 4) not planning to relocate out of Blantyre District in the next 12 months.

To assess the capacity of PWLE to provide informed consent, research assistants (RA) administered a structured comprehension checklist to assess understanding, information retention, and decision-making. During this process, the RA would invite the potential participant to repeat back key information on the consent form, such as the purpose of the study, risks and benefits, and duration of involvement. If the participant could not satisfactorily complete the checklist, the study staff member would speak to the legally authorized representative (LAR) to obtain consent. If the LAR did not accompany the potential participant, the RA would speak with the person who did accompany the potential participant to identify the LAR. In such a case, the LAR would be invited to provide informed consent on the participant’s behalf following standard IRB procedures.

Eligible and willing individuals provided IRB-approved written informed consent. Sixty people with psychosis and 118 caregivers enrolled. After completing a baseline survey, participants, along with their caregivers, were randomized 1:1 to receive enhanced usual care or the adapted CBR intervention. Participants and caregivers in both arms completed interviewer-administered follow-up research interviews at approximately 6 and 12 months.

### Intervention

Participants who were randomized to the intervention arm received the adapted CBR intervention delivered by the QECH clinical team of psychiatric nurses. CBR consists of nurse-delivered home visits over approximately 12 months with decreasing intensity as determined by the nursing team. In the initial phase, which focused on stabilization, weekly visits occurred for 2–4 months. During the intermediate phase, focused on rehabilitation, biweekly visits occurred for 4–6 months. In the final phase, focused on the sustainability of lessons learned and transition to independence, visits occurred monthly for 3–4 months.

The logistics of each phase were individualized by the nurses to address the participants’ needs from initial presentation and response to the intervention. Individualized modules to meet these needs could include sessions on mental health, day-to-day functioning, and rehabilitation. Intervention content was delivered with the PWLE and/or caregiver as appropriate to the module. Notably, one module for all participants during the intermediate phase focused on stigma and included material explaining different forms of stigma, encouraging positive coping, and empowering individuals to combat negative stereotypes. The module included exercises drawn from the What Matters Most framework, which theorizes that stigma is felt most acutely when someone is unable to participate in activities that “matter most” to them [37]. The goal of this module was to reduce internalized stigma among PWLE and reduce enacted stigma perpetrated by family members through improved knowledge and reduced anticipated stigma.

Participants randomized to enhanced usual care received no home-based services and continued to receive their standard clinical care at QECH. For these participants, the research team enhanced usual care through the provision of a summary of the symptomatology and disability scales, along with any relevant clinical recommendations to the clinical team.

### Measures

#### Internalized stigma.

Internalized stigma among people living with psychosis was measured with the Internalized Stigma of Mental Illness Inventory (ISMI)-10. The ISMI-10 is derived from the full ISMI, a 29-item Likert scale that is widely used to measure internalized stigma [38–40]. The ISMI-29 has five subscales measuring Alienation, Stereotype Endorsement, Perceived Discrimination, Social Withdrawal, and Stigma Resistance. The ISMI-10 includes items from each subscale that have been developed and psychometrically validated across contexts [39,40]. The ISMI-10 uses a 4-point Likert scale (‘strongly disagree’ = 1 to ‘strongly agree’ = 4) with total scores ranging from 10-40. Items 2 and 9 were reverse-coded as they represent stigma resilience. A continuous measure of the ISMI-10 total score was used in this analysis.

Internalized stigma among caregivers was measured with the 17-item Parents’ Internalized Stigma of Mental Illness Inventory (PISMI) [41]. The PISMI is also derived from the ISMI-29 and includes items adapted to capture aspects of the discrimination experience, social withdrawal and alienation, and stereotype endorsement subscales relevant to parents of children with severe mental illness [41]. The scale uses a 4-point Likert scale (‘strongly disagree’ = 1 to ‘strongly agree’ = 4) with scores ranging from 17-68. No items were reverse-coded as no stigma resilience questions are included. A continuous measure of the 17-item PISMI scale was used in this analysis.

#### Baseline predictors of internalized stigma.

Guided by our conceptual model, variables considered as predictors of internalized stigma among PWLE included measures of anticipated stigma, experienced stigma, psychosis severity, and various demographic variables.

Experienced stigma, anticipated stigma, and resilience among PWLE were assessed with the 34-item Discrimination and Stigma Scale (DISC-12) [42]. The DISC-12 uses a 4-point Likert scale (‘not at all’ = 0 to ‘a lot’ = 3). The DISC-12 has 4 subscales: unfair treatment (experienced stigma), stopping yourself from doing things because of conditions (anticipated stigma), overcoming stigma and discrimination (resilience), and positive treatment. Each subscale is evaluated independently by calculating the mean score and counting the total number of responses from 1-3, excluding items that received a ‘not at all’ response. No items are reverse-coded. We calculated the mean score and count for each subscale. As subscales are in different directions, a total score is not typically reported.

Based on the formative work [32], the study team designed a 5-item scale to evaluate family-perpetuated enacted stigma. This scale uses a 4-point Likert scale (‘strongly disagree’ = 1 to ‘strongly agree’ = 4), with total scores ranging from 5-20. Questions included “I am excluded from family events,” “When I’m having active symptoms, my family will use binds to control me,” “My family becomes unnecessarily angry with me,” “My family doesn’t respect me,” and “My family doesn’t allow me to have my own independence.”

Problematic or interfering psychosis symptoms were measured by the PANSS [43,44]. This scale uses a 7-point Likert scale denoting symptom severity from ‘absent’ (1) to ‘extreme’ (7), with scores ranging from 30 to 210. Total scores and binary cut-off scores (PANSS 58+) were used. Demographic variables assessed at baseline included age, sex, marital status, and education.

Data collection from caregivers was much more limited than from participants and only included demographic variables as potential predictors of internalized stigma.

#### Outcomes of internalized stigma.

Variables considered as possible outcomes of internalized stigma among PWLE included measures of overall functioning, quality of life, and mental health outcomes.

Overall functioning was measured by the 12-item World Health Organization Disability Assessment Schedule (WHODAS) [45]. The WHODAS uses a 5-point Likert scale (‘None’ = 1 to ‘Extreme or cannot do’ = 5), with scores ranging from 12-60. Quality of life was measured by the Short-Form 8 (SF-8) [46]. Normed total scores were used, with higher scores indicating better health.

The 9-item Patient Health Questionnaire (PHQ-9) was used to measure depressive symptoms [47]. This scale uses a 4-point Likert scale (‘0 days’ = 0 to ‘13 or 14 days’ = 3) to assess the frequency of presence of each of the nine core symptoms of a depressive episode in the prior two weeks, with total scores ranging from 0-27. We analyzed depressive symptoms using a continuous and binary (≥5 cut-off) measure of the PHQ-9.

The 7-item Generalized Anxiety Disorder scale (GAD-7) was used to measure anxiety symptoms [48]. GAD-7 uses a 4-point Likert scale (‘0 days’ = 0 to ‘13 or 14 days’ = 3) to assess the frequency of presence of each of the seven core symptoms of anxiety in the prior two weeks, with total scores ranging from 0-21. We analyzed anxiety symptoms using a continuous and binary (≥5) cut-off of the GAD-7.

### Statistical analysis

#### Exploratory factor analysis.

We performed an exploratory factor analysis (EFA) to identify the structure and dimensionality of all stigma scales. We calculated Cronbach’s alpha for each scale and conducted an EFA to assess whether each scale or subscale loaded on a single factor. We examined the number of eigenvalues greater than 1 and whether individual items loaded on the primary factor in the expected direction with approximately equal loadings. Values of Cronbach’s alpha greater than 0.7 were considered to be acceptable.

#### Demographics.

We described baseline sociodemographic, mental health, and functional measures using means (SD) for continuous variables and frequencies (percentages) for categorical variables.

#### Stigma over time and by arm.

We present descriptive statistics on internalized, anticipated, and enacted stigma at each time point (baseline, 6, and 12 months) using cross-sectional means and standard deviations (SD) at each time point. Although intervention effects on stigma were not of primary interest in this paper, we also descriptively report means of each measure by arm as well as mean differences between arms with 95% confidence intervals (CI) at each time point. Since the parent pilot study was not designed to be powered to detect effects with statistical significance tests, no testing was done.

#### Predictors of internalized stigma.

The selection of predictors was guided by our conceptual model (Fig 1). The goal of this portion of the analysis was descriptive rather than causal. The use of a multivariable predictive model in this case, with the inclusion of multiple explanatory variables of interest in the same model, is subject to the Table 2 Fallacy, making the majority of the regression coefficients uninterpretable [49]. Therefore, bivariable associations between each predictor and internalized stigma are reported.

To evaluate continuous baseline predictors of internalized stigma among PWLE and caregivers, we calculated bivariable correlations between baseline predictors and baseline ISMI total score. Correlation coefficients with an absolute value between 0 and 0.1 were considered to be approximately null; between 0.1 and 0.3 were considered to be weak associations, 0.3 and 0.7 were considered to be moderate associations, and greater than 0.7 were considered to be strong associations [50].

For binary and categorical baseline predictors, we calculated the mean difference in baseline ISMI total score between groups, calculated from a bivariable linear regression model.

To explore potential associations between changes in continuous predictors and changes in internalized stigma over the study period, we used generalized estimating equations for repeated outcomes clustered on the individual participant. We regressed the absolute change in each predictor from time *t-1* to time *t* on the change in internalized stigma score over the same interval, with two observations per individual (baseline to 6 months, and 6 months to 12 months). All models were adjusted for study arm.

Absolute change was calculated by subtracting a participant’s scale score at time *t* from their score at time *t-1*. All variables used in analyses over time were standardized to interpret regression coefficients consistently with the correlation coefficients.

#### Outcomes of internalized stigma.

We then explored potential associations between internalized stigma over the study period and our hypothesized outcomes, as guided by our conceptual model (Fig 1). We used a directed acyclic graph to identify potential confounders of the relationship between internalized stigma and our outcomes. We identified our adjustment set as sex, education, employment, and baseline psychosis severity. The adjustment set additionally included study arm for all analyses involving post-baseline values of or changes in stigma.

First, we used separate linear regression models to estimate the adjusted association between internalized stigma and each outcome at baseline. Next, we used generalized estimating equations for repeated outcomes to estimate the adjusted association between baseline internalized stigma and each outcome over follow-up, including 6-month and 12-month observations. Finally, we again used generalized estimating equations for repeated outcomes to estimate the adjusted association between change in internalized stigma from time *t-1* to time *t* and the value of the outcome at time *t*, with *t* representing both 6- and 12-month observations. All variables used in outcome analyses were standardized to interpret regression coefficients consistently with the correlation coefficients.

All measures of association are reported with 95% CI to show precision, direction, and magnitude of effect that are not captured by p-values. Percentile-based bootstrapped 95% CI based on 2,000 replications are reported for correlation coefficients.

All analyses were done in Stata 18.0 [51].

## Results

### People with lived experience

Between December 5^th^, 2023, and July 30^th^, 2024, 60 people with lived experience were enrolled and completed the baseline survey. Their mean (SD) age was 38.1 (12) years. The majority were male (76.7%), never married (63.3%), and had completed secondary school (48.3%). Eighty-five percent had CGI scores greater than 3, 50% had PANSS scores greater than 58, and about 3% had WHODAS scores greater than 35. Over a third had PHQ-9 scores greater than 5 (36.7%) and a fourth had GAD-7 scores greater than 5 (26.7%) (Table 1).

**Table 1:** Demographics of PWLE and caregivers. Values are presented as mean (SD) for continuous variables and n (%) for categorical variables. SD = standard deviation; N = number of participants. The study sample includes all PWLE (N = 60) and their caregivers (N = 118) participating in a community-based rehabilitation intervention in Malawi. Participants were recruited between December 5^th^, 2023, and July 30^th^, 2024. Alcohol use and smoking variables were assessed using relevant DSM-5 items. These items were added one-third of the way through baseline interviews, resulting in 21 missing observations. PWLE age range: 20-69, Caregiver age range: 18-84, PWLE GAD-7 total score range: 0-17, PWLE PHQ-9 total score range: 0-21, PWLE SF-8 total score range: 21.5-63.4, PWLE PANSS total score range: 30-133, PWLE WHODAS total score range: 12-56.

	PWLE	Caregivers
	N = 60	N = 118
Age (years), mean (SD)	38.1 (12.4)	43.7 (15.5)
Sex		
Female	14 (23.3%)	76 (64.4%)
Male	46 (76.7%)	42 (35.6%)
Marriage		
Never Married	38 (63.3%)	20 (16.9%)
Currently Married	13 (21.7%)	69 (58.5%)
Previously Married	9 (15.0%)	29 (24.6%)
Education		
No Schooling	3 (5.0%)	5 (4.2%)
Standard 5	4 (6.7%)	12 (10.2%)
Standard 8	13 (21.7%)	33 (28.0%)
Secondary School	29 (48.3%)	48 (40.7%)
Postsecondary	11 (18.3%)	20 (16.9%)
Employment		
Employed/Actively contributing	30 (50.0%)	
Unemployed able to work	8 (13.3%)	
Unemployed unable to work	3 (5.0%)	
Other/Refused	19 (31.7%)	
Drinking 4 + drinks in a single day*		
None	36 (92.3%)	
Less than a day or two	1 (2.6%)	
Several days	2 (5.1%)	
Smoking*		
None	34 (87.2%)	
Several days	2 (5.1%)	
More than 1/2 days	1 (2.6%)	
Nearly every day	2 (5.1%)	
GAD Total Score, mean (SD)	3.3 (3.9)	
GAD-7 Score 5+	16 (26.7%)	
PHQ Total Score, mean (SD)	4.5 (4.3)	
PHQ-9 Score 5+	22 (36.7%)	
SF-8 Total Score, mean (SD)	47.2 (8.7)	
CGI 3+ Plus	51 (85.0%)	
PANSS Total Score, mean (SD)	63.9 (26.9)	
PANSS 58+	30 (50.0%)	
WHODAS Total Score, mean (SD)	19.9 (7.2)	
WHODAS Total 35+	2 (3.3%)	

*21 missing values, DSM-V was added a third of the way through baseline interviews.

In our exploratory factor analysis, stigma scales generally demonstrated adequate internal consistency, with three exceptions. Notably, the DISC-12 subscale of overcoming stigma had a very low alpha of 0.12. This may be due to the subscale only having two items (Table 2). EFA generally supported a single-factor structure for each of the stigma scales.

**Table 2:** Internalized, anticipated, and experienced stigma over time among PWLE. Values are presented as mean (SD) scores at each time point. Cronbach’s alpha coefficients reflect the internal consistency and reliability of each stigma scale at baseline. Sample sizes were N = 60 at baseline, N = 59 at 6 months, and N = 58 at 12 months. Stigma score range at baseline: Internalized Stigma: 20-30, DISC-12 Unfair Treatment: 0-28, DISC-12 Stopped Yourself: 0-12, DISC-12 Overcoming Stigma Discrimination: 0-6, DISC-12 Positive Treatment: 0-15, Family-Enacted Stigma: 5-15.

	Cronbach’s alpha	Baseline	6 months	12 months
Mean (SD)		(N = 60)	(N = 59)	(N = 58)
Internalized Stigma	0.72	25.2 (2.3)	24.8 (2.4)	23.8 (2.4)
DISC-12 Unfair Treatment	0.86	3.6 (5.9)	1.7 (4.1)	1.8 (4.5)
DISC-12 Stopped Yourself	0.62	2.3 (2.9)	1.7 (2.4)	1.1 (2.2)
DISC-12 Overcome Stigma Discrimination	0.12	2.6 (1.5)	2.3 (1.4)	2.6 (1.6)
DISC-12 Positive Treatment	0.61	10.1 (3.4)	9.8 (4.0)	10.9 (3.6)
Family-Enacted Stigma	0.79	11.1 (2.5)	11.5 (2.0)	10.6 (1.8)

We examined how internalized stigma, anticipated stigma, and experienced stigma changed over time. Internalized stigma, measured by the ISMI scale, decreased over the study period with a mean (SD) of 25.2 (2.3) at baseline, 24.8 (2.4) at 6 months, and 23.8 (2.4) at 12 months. Anticipated stigma, measured by the DISC-12 subscale of stopping yourself from engaging in activities, decreased over time with a mean of 2.3 (2.9) at baseline, 1.7 (2.4) at 6 months, and 1.1 (2.2) at 12 months. Experienced stigma, measured by the DISC-12 subscale of unfair treatment, decreased from 3.6 (5.9) at baseline to 1.7 (4.1) at 6 months, then slightly increased to 1.8 (4.5) at 12 months. Experienced stigma measured by family-enacted stigma increased from 11.1 (2.5) at baseline to 11.5 (2.0) at 6 months, then decreased to 10.6 (1.8) at 12 months (Table 2 and Fig 2).

**Fig 2:**
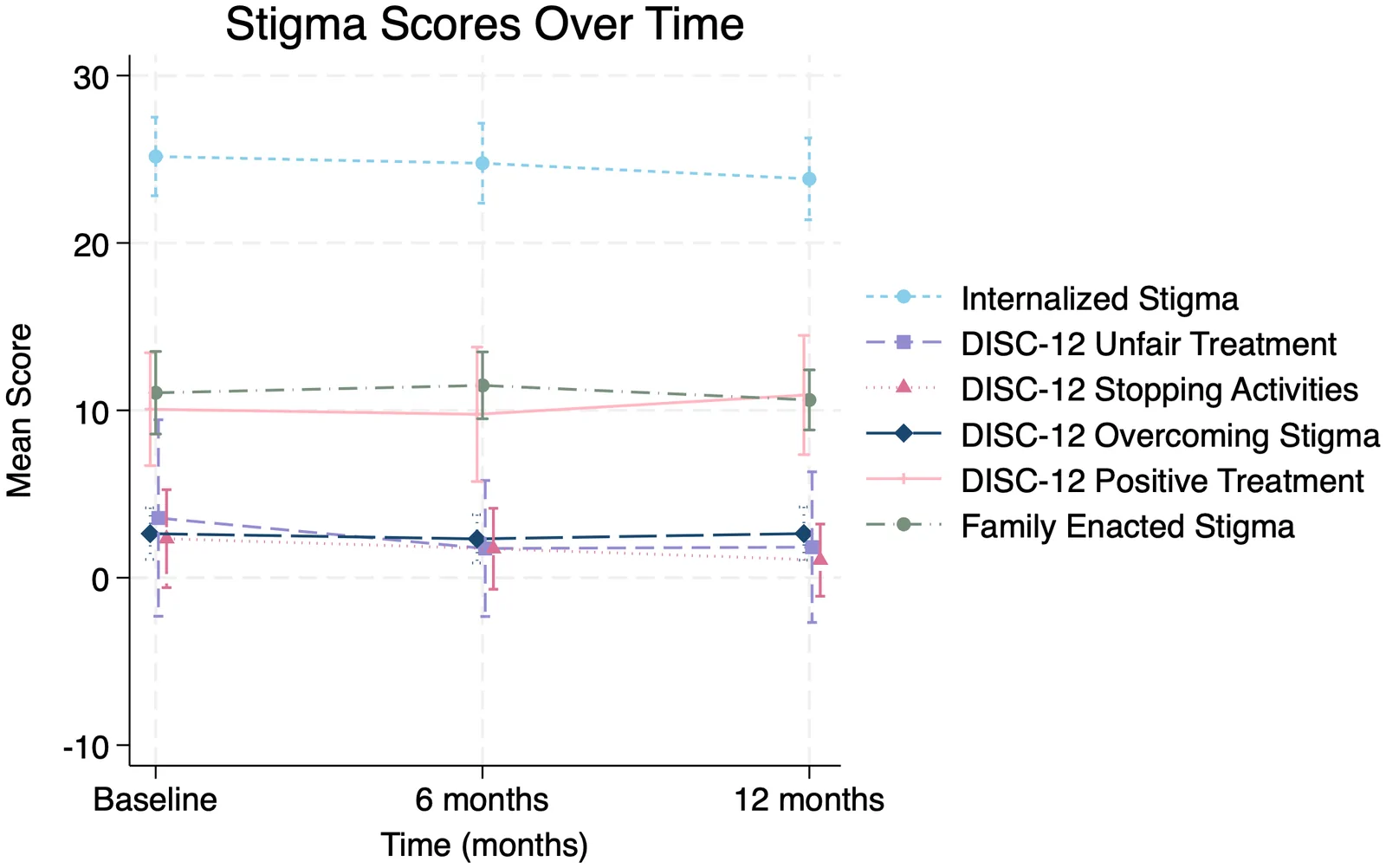
Internalized, anticipated, and experienced stigma over time among PWLE. Mean stigma scores over the study period, with error bars representing the mean plus/minus one standard deviation (SD). Mean values in the figure correspond to the reported mean (SD) values in Table 2.

In general, there were no substantively meaningful or statistically significant differences in internalized stigma, anticipated stigma, and experienced stigma change over time by arm, with one exception (S1 Table). Among those receiving the intervention, there was a -0.97 (-1.88, -0.05) difference in mean family-enacted stigma scores at 12 months compared to those receiving usual care (S2 Table).

We evaluated mean differences and associations between ISMI scores and potential predictors over time to understand factors contributing to baseline and changing ISMI scores (Fig 1). At baseline, hypothesized binary and categorical predictors, including PANSS scores greater than 58, sex, employment, marital status, and education, were not associated with mean baseline ISMI scores (Table 3). Additionally, hypothesized continuous predictors, including DISC-12 subscales of anticipated stigma, overcoming stigma, and positive treatment, were not associated with ISMI baseline scores. Age, PANSS total score, and DISC-12 subscale of unfair treatment were weakly associated with baseline ISMI scores. Higher family-enacted stigma scores were moderately associated with higher ISMI scores (correlation coefficient = 0.34; 0.13, 0.53) at baseline (Table 4).

**Table 3:** Binary and categorical predictors of ISMI among PWLE at baseline. Values presented are the mean (SD) for ISMI scores within each variable category. Mean differences represent the unadjusted difference in ISMI total score compared to the defined reference group (REF) for each predictor. Mean differences were estimated using bivariate regression models. SD = standard deviation; SE = standard error; CI = confidence interval.

	ISMI Score Mean (SD)	Mean Difference (SE)	95% CI
Sex			
Female	25.4 (2.3)	0.3 (0.7)	(-1.1, 1.8)
Male	25.1 (2.4)	REF	REF
PANSS 58+			
0-57	24.7 (2.2)	REF	REF
58+	25.6 (2.4)	0.9 (0.6)	(-0.3, 2)
Employed/Actively contributing			
No	25.2 (0.4)	REF	REF
Yes	25.1 (0.5)	-0.1 (0.6)	(-1.3, 1.1)
Marital Status			
Never Married	25.2 (2.2)	REF	REF
Currently Married	25.4 (2.6)	0.2 (0.8)	(-1.3, 1.8)
Previously Married	24.9 (2.8)	-0.3 (0.9)	(-2.0, 1.8)
Education			
No schooling	25.3 (3.8)	-0.1 (1.5)	(-3.0, 2.8)
Standard 5	24.3 (3.3)	-1.2 (1.3)	(-3.8, 1.4)
Standard 8	25.2 (2.5)	-0.2 (0.8)	(-1.8, 1.4)
Secondary School	25.4 (2.3)	REF	REF
Postsecondary	24.6 (1.7)	-0.8 (0.8)	(-2.5, 0.9)

Total sample N = 60.

**Table 4:** Continuous predictors of ISMI among PWLE at baseline. Values presented represent unadjusted bivariable correlation coefficients with corresponding 95% confidence intervals (CI). Correlation coefficients were estimated using Pearson correlations. 95% CI were obtained using percentile-based bootstrapping with 2,000 replications.

	Correlation Coefficient	95% CI
**Continuous Predictors**		
Age (years)	-0.15	(-0.40, 0.13)
PANSS Total Score	0.18	(-0.06, 0.41)
DISC-12 Unfair Treatment Score	0.14	(-0.10, 0.39)
DISC-12 Stopped Yourself Score	0.07	(-0.22, 0.34)
DISC-12 Overcoming Stigma Discrimination Score	-0.03	(-0.32, 0.25)
DISC-12 Positive Treatment Score	0.07	(-0.20, 0.32)
Family-Enacted Stigma	0.34	(0.13, 0.53)

Total N = 60.

We evaluated associations between changes in hypothesized predictor variables and changes in ISMI scores over time, adjusted for arm. Changes in PANSS total score and DISC-12 subscales of unfair treatment, overcoming stigma, and positive treatment were not associated with changes in ISMI scores (Table 5). Greater change in family-enacted stigma scores (0.24; 0.04, 0.43) and anticipated stigma scores (0.14; 0.02, 0.26) were weakly associated with greater change in ISMI scores over time (Table 5).

**Table 5:** Associations between the standardized absolute change in continuous predictors and the standardized absolute change in ISMI among PWLE. Regression coefficients with corresponding 95% confidence intervals (CI) were generated by a generalized estimating equation (GEE) for repeated outcomes clustered by individual participant. For each predictor, we regressed the standardized absolute change from time t-1 to time t on the standardized change in internalized stigma score over that same interval. Absolute change was calculated by subtracting the participant’s scale score at time t from their score at time t-1. Then absolute change values were standardized. All models were adjusted for study arm. Total N for GEE models = 117, at 6 months, N = 59, and N = 58 at 12 months.

	Change in ISMI Score	
	β	95% CI
**Change in Predictor Score**		
PANSS Total Score	0.08	(-0.08, 0.25)
DISC-12 Unfair Treatment Score	0.10	(-0.06, 0.27)
DISC-12 Stopped Yourself Score	0.14	(0.02, 0.26)
DISC-12 Overcoming Stigma Discrimination Score	0.07	(-0.14, 0.28)
DISC-12 Positive Treatment Score	0.08	(-0.12, 0.27)
Family-Enacted Stigma	0.24	(0.04, 0.43)

Total N = 59 at 6 months.

Total N = 58 at 12 months.

To explore the impact of baseline internalized stigma and changes in internalized stigma on anticipated outcomes, we evaluated associations between ISMI scores and hypothesized mental health and functional outcomes over time (Fig 1). At baseline, ISMI scores were weakly associated with depressive symptoms (0.14; -0.08, 0.35) and anxiety symptoms (0.11; -0.08, 0.31). Over time, baseline ISMI scores were weakly associated with depressive symptoms (-0.11; -0.25, 0.03), anxiety symptoms (-0.13; -0.30, 0.04), functionality (0.22; -0.10, 0.53), and quality of life (-0.14; -0.34, 0.07) (Table 6).

**Table 6:** Associated outcomes of ISMI across the study period. Regression coefficients with corresponding 95% confidence intervals (CI) were generated by the following models. Baseline associations: separate adjusted linear regression models to estimate the baseline association between standardized internalized stigma and standardized predicted outcome. Baseline ISMI associated with follow-up outcomes: generalized estimating equations (GEE) for repeated outcomes were used to estimate the association between standardized baseline internalized stigma and each standardized outcome over follow-up, including 6-month and 12-month observations. Change in ISMI associated with concurrent outcomes: GEE for repeated outcomes was used to estimate the association between the standardized change in internalized stigma from time t-1 to time t and the standardized value of the outcome at time t, with t representing both 6- and 12-month observations. Adjustment sets for all models included education, employment, sex, and psychosis severity. Arm was adjusted for in models over time. Absolute change was calculated by subtracting the participant’s scale score at time t from their score at time t-1. Absolute change was then standardized. Positive coefficients indicate higher ISMI scores or increases in ISMI are associated with higher outcome scores; negative coefficients indicate inverse associations. A baseline N = 60, at 6 months N = 59, and at 12 months N = 58. GEE models had N = 117 observations reflecting the sample size at 6 and 12 months.

	Baseline association		Baseline ISMI associated with follow-up outcomes		Change in ISMI associated with concurrent outcomes	

	β	95% CI	β	95% CI	β	95% CI
PHQ-9	0.14	(-0.08, 0.35)	-0.11	(-0.25, 0.03)	0.06	(-0.11, 0.23)
GAD-7	0.11	(-0.08, 0.31)	-0.13	(-0.30, 0.04)	0.13	(0.01, 0.24)
WHODAS	0.06	(-0.10, 0.22)	0.22	(-0.10, 0.53)	-0.04	(-0.13, 0.05)
SF-8	0.06	(-0.19, 0.31)	-0.14	(-0.34, 0.07)	0.01	(-0.15, 0.16)

Total N = 60 at baseline.

Total N = 59 at 6 months.

Total N = 58 at 12 months.

At 6 and 12 months, we evaluated associations between changes in ISMI scores and hypothesized outcomes. Greater increases in ISMI scores were associated with higher anxiety symptoms over time (0.13; 0.01, 0.24). Changes in ISMI scores were not associated with other hypothesized outcomes (Table 6).

### Caregivers

One hundred eighteen caregivers completed the baseline survey between December 6^th^, 2023, and July 30^th^, 2024. The average age was 43.7 years, and the majority were female (64.4%), currently married (58.5%), and had completed secondary school (40.7%) (Table 1).

Internalized stigma decreased among caregivers over the study period, with a mean and SD of 41.7 (5.4) at baseline, 40.1 (5.0) at 6 months, and 38.8 (5.0) at 12 months. Hypothesized predictors, including sex, age, marital status, and education, were not associated with mean baseline ISMI scores (Table 7).

**Table 7:** Binary and categorical predictors of ISMI among caregivers at baseline. Values presented are the mean (SD) for ISMI scores within each variable category. Mean differences represent the unadjusted difference in ISMI total score compared to the defined reference group (REF) for each predictor. Mean differences were estimated using bivariate regression models. SD = standard deviation; SE = standard error; CI = confidence interval.

	ISMI Score Mean (SD)	Mean Difference (SE)	95% CI
Sex			
Female	42.3 (5.5)	1.6 (1.0)	(-0.4, 3.6)
Male	40.6 (5.1)	REF	REF
Age			
Below the median	42.1 (5.8)	0.8 (1.0)	(-1.2, 2.7)
Above the median	41.3 (5.0)	REF	REF
Marital Status			
Never Married	40.5 (4.7)	-1.3 (1.4)	(-4.0, 1.4)
Currently Married	41.7 (5.5)	REF	REF
Previously Married	42.4 (5.6)	0.7 (1.2)	(-1.6, 3.1)
Education			
No Schooling	43.8 (5.0)	2.5 (2.4)	(-2.3, 7.3)
Standard 5	44.7 (4.1)	3.4 (1.7)	(0.0, 6.7)
Standard 8	42.7 (6.0)	1.4 (1.2)	(-0.9, 3.7)
Secondary School	41.3 (5.2)	REF	REF
Postsecondary	38.6 (4.2)	-2.7 (1.4)	(-5.4, 0.0)

Total sample N = 118.

## Discussion

To our knowledge, this is the first study to examine the predictors and outcomes of internalized stigma among people living with psychosis and their caregivers in Malawi. We found that PWLE and caregivers reported moderate levels of internalized stigma on the ISMI, suggesting internalized stigma experiences are common in this population. Among PWLE, participants reported moderate levels of family-enacted stigma, suggesting that family-enacted stigma is prevalent in this setting. Over the course of the study period, anticipated stigma, experienced stigma, and internalized stigma slightly decreased, with only family-enacted experienced stigma differing by arm at 12 months.

While the primary purpose of this analysis was not to examine stigma outcomes by study arm, the greater decrease in family-enacted stigma in the intervention compared to the usual care arm at 12 months is notable. The CBR intervention included a stigma reduction module delivered during the intermediate phase of the intervention, suggesting that the module may have led to subsequent decreases in family-enacted stigma at 12 months. These findings suggest improved knowledge of psychosis and stigma processes may reduce enacted stigma perpetrated by family members.

When exploring predictors of internalized stigma, we found that greater family-enacted stigma was moderately associated with higher internalized stigma among PWLE at baseline. We also found that, over time, a greater change in family-enacted stigma and anticipated stigma were weakly associated with a greater change in internalized stigma. These results highlight the role of family treatment and anticipated stigma in the adoption of stigmatizing beliefs. In combination with the moderate prevalence of family-enacted stigma, anticipated stigma, and the improvement in family-enacted stigma in the intervention group, our findings support a potential pathway to intervene on family-enacted stigma and anticipated stigma to subsequently reduce internalized stigma among PWLE. In contrast, we found no strong predictors of internalized stigma among caregivers. Importantly, these associations cannot be interpreted causally, given the lack of causal modeling and potential for confounding that may influence results.

Our results directly align with previous work in the region that describes how experienced stigma from family members and anticipating stigma can lead to the adoption of stigmatizing beliefs [16,32]. In contrast, other hypothesized predictors, and changes in predictors, such as psychosis severity and sociodemographic variables [31], were not associated with internalized stigma in this population. These findings differ from prior studies that have associated having less education and worse psychosis severity with more internalized stigma [31,32,52]. Results may reflect differences within the Malawian context, suggesting that family-enacted stigma and changes in anticipated stigma may be more important for internalizing stigmatizing beliefs compared to other predictors. While psychometric properties were adequate across our stigma constructs, additional work must be done with larger sample sizes to confirm the measurement accuracy of all study constructs.

When exploring outcomes of internalized stigma, higher internalized stigma scores were weakly associated with worse depressive and anxiety symptoms at baseline. These findings align with the global and local literature, associating greater internalized stigma with higher depressive and anxiety symptoms [15,16,34,52,53]. Over time, higher baseline internalized stigma scores were weakly associated with hypothesized mental health and functional outcomes. Results suggest that higher baseline internalized stigma was weakly associated with fewer depressive and anxiety symptoms over time. In contrast, greater increases in internalized stigma were weakly associated with greater increases in anxiety symptoms over time, as hypothesized. These conflicting results may be due to the low overall level of depressive and anxiety symptoms, especially after baseline. Additionally, results suggest that a change measure of internalized stigma may more accurately capture relationships over time. While outcome associations were adjusted for confounders, there may be residual, unmeasured confounding that is not captured in our confounder set.

Overall, this study provides evidence on the prevalence, predictors, and outcomes of internalized stigma among people with lived experience of psychosis and their caregivers. Our findings underscore that anticipated, experienced, and internalized stigma are prevalent. Additionally, results highlight the importance of family-enacted stigma and anticipated stigma on subsequent internalized stigma among PWLE. Future work should explore the stability of stigma measurement across cultural contexts and continue to explore mechanisms between predictors and outcomes of internalized stigma to inform stigma reduction strategies. In conclusion, our findings provide a foundation to further explore ways to reduce stigma among people with lived experience of psychosis and their caregivers in low-resource settings to promote recovery and well-being.

## Supporting information

S1 Checklist Inclusivity in global research. (DOCX)

S1 Table Internalized, anticipated, and experienced stigma over time by arm assignment among PWLE. Values are presented as mean (SD) scores at each time point. Total sample sizes were N = 60 at baseline, N = 59 at 6 months, and N = 58 at 12 months.
(DOCX)

S2 Table Mean difference in stigma scores by arm. Values are presented as mean differences with 95% confidence intervals (CI) at each time point. Mean differences at each time point were calculated by using a linear regression model with stigma score at that time point as the outcome and arm assignment at baseline as the predictor. Total sample sizes were N = 60 at baseline, N = 59 at 6 months, and N = 58 at 12 months.
(DOCX)

## References

[1] Zhan Z, Wang J, Shen T. Results of the Global Burden of Disease study for schizophrenia: Trends from 1990 to 2021 and projections to 2050. Front Psychiatry. 2025;16:1629032. doi: 10.3389/fpsyt.2025.1629032 40980052 PMC12447577

[2] Kane JC, Elafros MA, Murray SM, Mitchell EMH, Augustinavicius JL, Causevic S, et al. A scoping review of health-related stigma outcomes for high-burden diseases in low- and middle-income countries. BMC Med. 2019;17(1):17. doi: 10.1186/s12916-019-1250-8 30764819 PMC6376728

[3] Rössler W. The stigma of mental disorders: A millennia-long history of social exclusion and prejudices. EMBO Rep. 2016;17(9):1250–3. doi: 10.15252/embr.201643041 27470237 PMC5007563

[4] Link BG, Phelan JC. Conceptualizing stigma. Annu Rev Sociol. 2001;27:363–85.

[5] Quinn DM, Williams MK, Weisz BM. From discrimination to internalized mental illness stigma: The mediating roles of anticipated discrimination and anticipated stigma. Psychiatr Rehabil J. 2015;38(2):103–8. doi: 10.1037/prj0000136 25844910 PMC4469573

[6] Kruse EA, Dodell-Feder D. Schizophrenia spectrum stigma in healthcare: A systematic review. Front Psychiatry. 2025;16:1648957. doi: 10.3389/fpsyt.2025.1648957 40927017 PMC12414995

[7] Shibre T, Negash A, Kullgren G, Kebede D, Alem A, Fekadu A, et al. Perception of stigma among family members of individuals with schizophrenia and major affective disorders in rural Ethiopia. Soc Psychiatry Psychiatr Epidemiol. 2001;36(6):299–303. doi: 10.1007/s001270170048 11583460

[8] Monnapula-Mazabane P, Petersen I. Mental health stigma experiences among caregivers and service users in South Africa: A qualitative investigation. Curr Psychol. 2023;42(11):9427–39. doi: 10.1007/s12144-021-02236-y 34465971 PMC8396139

[9] Favina A, Hobe N, Muwanguzi M, Munaru G, Rubega A, Lutasingwa D, et al. Affiliate stigma and associated factors among informal caregivers of people with mental illness in southwestern Uganda: A multi-center cross-sectional study. PLOS Ment Health. 2025;2(4):e0000132. doi: 10.1371/journal.pmen.0000132 41661948 PMC12798226

[10] Ae-Ngibise KA, Doku VCK, Asante KP, Owusu-Agyei S. The experience of caregivers of people living with serious mental disorders: A study from rural Ghana. Glob Health Action. 2015;8:26957. doi: 10.3402/gha.v8.26957 25967587 PMC4429259

[11] Nsereko JR, Kizza D, Kigozi F, Ssebunnya J, Ndyanabangi S, Flisher AJ, et al. Stakeholder’s perceptions of help-seeking behaviour among people with mental health problems in Uganda. Int J Ment Health Syst. 2011;5:5. doi: 10.1186/1752-4458-5-5 21314989 PMC3050843

[12] Jahn DR, Leith J, Muralidharan A, Brown CH, Drapalski AL, Hack S, et al. The influence of experiences of stigma on recovery: Mediating roles of internalized stigma, self-esteem, and self-efficacy. Psychiatr Rehabil J. 2020;43(2):97–105. doi: 10.1037/prj0000377 31233321 PMC6928452

[13] Rüsch N, Corrigan PW, Wassel A, Michaels P, Larson JE, Olschewski M, et al. Self-stigma, group identification, perceived legitimacy of discrimination and mental health service use. Br J Psychiatry. 2009;195(6):551–2. doi: 10.1192/bjp.bp.109.067157 19949209 PMC2801828

[14] Rice WS, Crockett KB, Mugavero MJ, Raper JL, Atkins GC, Turan B. Association between internalized HIV-related stigma and HIV care visit adherence. J Acquir Immune Defic Syndr. 2017;76(5):482–7. 10.1097/QAI.0000000000001543 PMC5680126 28885270

[15] Turan B, Hatcher AM, Weiser SD, Johnson MO, Rice WS, Turan JM. Framing mechanisms linking HIV-related stigma, adherence to treatment, and health outcomes. Am J Public Health. 2017;107(6):863–9. 10.2105/AJPH.2017.303744 PMC5425866 28426316

[16] Fox AB, Earnshaw VA, Taverna EC, Vogt D. Conceptualizing and measuring mental illness stigma: The mental illness stigma framework and critical review of measures. Stigma Health. 2018;3(4):348–76. doi: 10.1037/sah0000104 30505939 PMC6261312

[17] Boyd JE, Adler EP, Otilingam PG, Peters T. Internalized Stigma of Mental Illness (ISMI) scale: A multinational review. Compr Psychiatry. 2014;55(1):221–31. doi: 10.1016/j.comppsych.2013.06.005 24060237

[18] Yanos PT, Lysaker PH, Roe D. Internalized stigma as a barrier to improvement in vocational functioning among people with schizophrenia-spectrum disorders. Psychiatry Res. 2010;178(1):211–3. doi: 10.1016/j.psychres.2010.01.003 20417973 PMC2914138

[19] Abdisa E, Fekadu G, Girma S, Shibiru T, Tilahun T, Mohamed H. Self-stigma and medication adherence among patients with mental illness treated at Jimma University Medical Center, Southwest Ethiopia. Int J Ment Health Syst. 2020;14:56. 10.1186/s13033-020-00391-6 PMC7391813 32760443

[20] Barnett BS, Kusunzi V, Magola L, Borba CPC, Udedi M, Kulisewa K, et al. Description of the inpatient population and care received at a psychiatric unit in Lilongwe, Malawi. Int J Cult Ment Health. 2018;11(4):574–82. doi: 10.1080/17542863.2018.1448424 32863863 PMC7450978

[21] Kaminga AC, Myaba J, Dai W, Liu A, Chilale HK, Kubwalo PF, et al. Association between referral source and duration of untreated psychosis in pathways to care among first episode psychosis patients in Northern Malawi. Early Interv Psychiatry. 2020;14(5):594–605. doi: 10.1111/eip.12885 31657157 PMC7496144

[22] Crabb J, Stewart RC, Kokota D, Masson N, Chabunya S, Krishnadas R. Attitudes towards mental illness in Malawi: A cross-sectional survey. BMC Public Health. 2012;12:541. doi: 10.1186/1471-2458-12-541 22823941 PMC3413535

[23] Kokota D, Stewart RC, Bandawe C, Chorwe-Sungani G, Liwimbi O, Mwale CM, et al. Pathways to care for psychosis in Malawi. BJPsych Int. 2023;20(4):84–9. doi: 10.1192/bji.2023.18 38029442 PMC10659844

[24] Sibande W, Chasweka D, Chidzalo K, Kokota D, Umar E, Amos A, et al. “They treat us like rabid dogs”: Stigma and discrimination as experienced by people living with psychosis and their caregivers in Malawi-A photovoice study. PLOS Ment Health. 2025;2(9):e0000435. doi: 10.1371/journal.pmen.0000435 41662043 PMC12798401

[25] Goffman E. Stigma: Notes on the management of spoiled identity. Englewood Cliffs, New Jersey: Prentice-Hall Inc.; 1963.

[26] de Wet A, Pretorius C. From darkness to light: Barriers and facilitators to mental health recovery in the South African context. Int J Soc Psychiatry. 2022;68(1):82–9. doi: 10.1177/0020764020981126 33307927

[27] Asher L, Fekadu A, Hanlon C, Mideksa G, Eaton J, Patel V, et al. Development of a community-based rehabilitation intervention for people with schizophrenia in Ethiopia. PLoS One. 2015;10(11):e0143572. doi: 10.1371/journal.pone.0143572 26618915 PMC4664267

[28] Chatterjee S, Patel V, Chatterjee A, Weiss HA. Evaluation of a community-based rehabilitation model for chronic schizophrenia in rural India. Br J Psychiatry. 2003;182:57–62. doi: 10.1192/bjp.182.1.57 12509319

[29] de Wet A, Swartz L, Chiliza B. Hearing their voices: The lived experience of recovery from first-episode psychosis in schizophrenia in South Africa. Int J Soc Psychiatry. 2015;61(1):27–32. doi: 10.1177/0020764014535753 24874119

[30] Yigit I, Modi RA, Weiser SD, Johnson MO, Mugavero MJ, Turan JM, et al. Effects of an intervention on internalized HIV-related stigma for individuals newly entering HIV care. AIDS. 2020;34 Suppl 1(Suppl 1):S73–82. doi: 10.1097/QAD.0000000000002566 32881796 PMC8075078

[31] Getinet Alemu W, Mwanri L, Due C, Azale T, Ziersch A. Predictors of internalised stigma among people with mental illness attending a psychiatry outpatient clinic in Ethiopia: Institution based cross sectional study. PLoS One. 2025;20(3):e0319458. doi: 10.1371/journal.pone.0319458 40100887 PMC11918445

[32] Stockton MA, Kramer J, Chienda J, Morrison AM, Tikhiwa HA, Sansbury G, et al. Applying the Health Stigma and Discrimination Framework to psychosis stigma in Malawi. PLOS Ment Health. 2025;2(5):e0000306. doi: 10.1371/journal.pmen.0000306 40548334 PMC12180428

[33] Egbe CO, Brooke-Sumner C, Kathree T, Selohilwe O, Thornicroft G, Petersen I. Psychiatric stigma and discrimination in South Africa: Perspectives from key stakeholders. BMC Psychiatry. 2014;14:191. doi: 10.1186/1471-244X-14-191 24996420 PMC4099203

[34] Drapalski AL, Lucksted A, Perrin PB, Aakre JM, Brown CH, DeForge BR, et al. A model of internalized stigma and its effects on people with mental illness. Psychiatr Serv. 2013;64(3):264–9. doi: 10.1176/appi.ps.001322012 23573532

[35] Nyblade L, Stockton MA, Giger K, Bond V, Ekstrand ML, Lean RM, et al. Stigma in health facilities: Why it matters and how we can change it. BMC Med. 2019;17(1):25. doi: 10.1186/s12916-019-1256-2 30764806 PMC6376713

[36] Alemu WG, Due C, Muir-Cochrane E, Mwanri L, Azale T, Ziersch A. Quality of life among people living with mental illness and predictors in Africa: A systematic review and meta-analysis. Qual Life Res. 2024;33(5):1191–209. doi: 10.1007/s11136-023-03525-8 37906348 PMC11045618

[37] Yang LH, Chen F-P, Sia KJ, Lam J, Lam K, Ngo H, et al. “What matters most:” A cultural mechanism moderating structural vulnerability and moral experience of mental illness stigma. Soc Sci Med. 2014;103:84–93. doi: 10.1016/j.socscimed.2013.09.009 24507914

[38] Ritsher JB, Otilingam PG, Grajales M. Internalized stigma of mental illness: Psychometric properties of a new measure. Psychiatry Res. 2003;121(1):31–49. doi: 10.1016/j.psychres.2003.08.008 14572622

[39] van Beukering IE, Bakker M, Bogaers RI, Janssens KME, Gürbüz S, Joosen MCW, et al. Psychometric properties of the Internalized Stigma of Mental Illness (ISMI-10) scale in a Dutch sample of employees with mental illness. BMC Psychiatry. 2022;22(1):662. 10.1186/s12888-022-04284-5 PMC9609266 36303128

[40] Boyd JE, Otilingam PG, Deforge BR. Brief version of the Internalized Stigma of Mental Illness (ISMI) scale: Psychometric properties and relationship to depression, self esteem, recovery orientation, empowerment, and perceived devaluation and discrimination. Psychiatr Rehabil J. 2014;37(1):17–23. doi: 10.1037/prj0000035 24660946

[41] Zisman-Ilani Y, Levy-Frank I, Hasson-Ohayon I, Kravetz S, Mashiach-Eizenberg M, Roe D. Measuring the internalized stigma of parents of persons with a serious mental illness: The factor structure of the parents’ internalized stigma of mental illness scale. J Nerv Ment Dis. 2013;201(3):183–7. doi: 10.1097/NMD.0b013e3182845d00 23443038

[42] Brohan E, Clement S, Rose D, Sartorius N, Slade M, Thornicroft G. Development and psychometric evaluation of the Discrimination and Stigma Scale (DISC). Psychiatry Res. 2013;208(1):33–40. doi: 10.1016/j.psychres.2013.03.007 23582210

[43] Kay SR, Fiszbein A, Opler LA. The positive and negative syndrome scale (PANSS) for schizophrenia. Schizophr Bull. 1987;13(2):261–76. doi: 10.1093/schbul/13.2.261 3616518

[44] Leucht S, Kane JM, Kissling W, Hamann J, Etschel E, Engel RR. What does the PANSS mean? Schizophr Res. 2005;79(2-3):231–8. 10.1016/j.schres.2005.04.008 15982856

[45] WHO. Measuring health and disability: Manual for WHO Disability Assessment Schedule (WHODAS 2.0). Geneva: World Health Organization; 2010.

[46] Ware JE Jr, Kosinski M, Dewey JE. How to score and interpret single-item health status measures: A manual for users of the SF-8™ Health Survey. Lincoln, RI: Quality Metric Incorporated; 2001.

[47] Kroenke K, Spitzer RL. The PHQ-9: A new depression diagnostic and severity measure. Psychiat Ann. 2002;32(9):509–15.

[48] Chibanda D, Verhey R, Gibson LJ, Munetsi E, Machando D, Rusakaniko S, et al. Validation of screening tools for depression and anxiety disorders in a primary care population with high HIV prevalence in Zimbabwe. J Affect Disord. 2016;198:50–5. doi: 10.1016/j.jad.2016.03.006 27011359

[49] Westreich D, Greenland S. The table 2 fallacy: Presenting and interpreting confounder and modifier coefficients. Am J Epidemiol. 2013;177(4):292–8. doi: 10.1093/aje/kws412 23371353 PMC3626058

[50] Akoglu H. User’s guide to correlation coefficients. Turk J Emerg Med. 2018;18(3):91–3. doi: 10.1016/j.tjem.2018.08.001 30191186 PMC6107969

[51] StataCorp. Stata 18. Statistical software. StataCorp LLC; 2023.

[52] Stangl AL, Earnshaw VA, Logie CH, van Brakel W, C. Simbayi L, Barré I, et al. The Health Stigma and Discrimination Framework: A global, crosscutting framework to inform research, intervention development, and policy on health-related stigmas. BMC Med. 2019;17(1):31. doi: 10.1186/s12916-019-1271-3 30764826 PMC6376797

[53] Hatzenbuehler ML, Phelan JC, Link BG. Stigma as a fundamental cause of population health inequalities. Am J Public Health. 2013;103(5):813–21. doi: 10.2105/AJPH.2012.301069 23488505 PMC3682466

